# Photodynamic larvicidal activity of magnesium chlorophyllin and magnesium chlorophyllin zinc oxide nanocomposite against *Spodoptera frugiperda* with non-target safety assessment

**DOI:** 10.1038/s41598-026-45022-1

**Published:** 2026-04-11

**Authors:** Hadeer M. Elshemy, Magda H. Rady, Shaimaa Mahmoud Farag Mahmoud, Tarek A. El-Tayeb, Ahmed A. El-Sayed, Moataz A. M. Moustafa, Ahmed Reda Ismaieel, Radwa G. Attia

**Affiliations:** 1 Entomology Department, Faculty of Science, Ain Shams University, Cairo, Egypt; 2 National Institute for Laser Enhanced Sciences (NILES), Cairo University, Giza, Egypt; 3 Photochemistry Department, Industrial Chemical Research Institute, National Research Centre, Dokki, Giza 12622 Egypt; 4 Economic Entomology and Pesticides Department, Faculty of Agriculture, Cairo University, Giza, 12613 Egypt; 5 Neuroethology Department, Max Planck Institute for Chemical Ecology, Hans-Knoell-Straße 8, 07745 Jena, Germany

**Keywords:** Photodynamic therapy, Integrated pest management, Oxidative stress, Ecological safety, Detoxification mechanisms, Biochemistry, Chemical biology, Environmental sciences, Plant sciences, Zoology

## Abstract

Chemical pesticides have been used as a reliable method of controlling pests on crops. However, their applications have led to the development of resistance, environmental problems, and risks to human health. Therefore, new chemicals in nanoform such as chlorophyll derivatives could be a good choice for pest control through the induction of reactive oxygen species, liberating singlet oxygen when they are excited by photo exposure. The present work evaluated the toxicity of magnesium chlorophyllin (Mg-Chl) and magnesium chlorophyllin zinc oxide (Mg-Chl–ZnO NC) nanocomposite on *Spodoptera frugiperda* (J.E. Smith) (Lepidoptera: Noctuidae)*.* Additionally, the biochemical changes of detoxifying enzymes, such as carboxylesterases (CXEs) and glutathione-*S*-transferases (GSTs), were investigated. In silico, molecular docking analysis was also performed to assess the binding affinities of the tested compounds to *CXE22* and *SfGSTe9*. Thus, the evaluation of potential photosensitizers against the second instar larvae of *Chrysoperla carnea* Stephens, as non-target insect and active predator of aphids, was conducted. The results showed that both Mg-Chl and Mg-Chl–ZnO NC have a high potency against *S. frugiperda* with LC_50_ = 0.59 and 0.49 mg L^−1^, respectively, after 12-h dark incubation and 5-h light exposure, while after 24 h, LC_50_ values after dark incubation were 0.51 and 0.31 mg L^−1^, respectively. Both tested compounds exhibited a strong binding affinity to *CXE22 and SfGSTe9*, leading to significant alterations in CXEs activity and GSTs activity in correspondence with the oxidative stress and contributing to larval mortality. In parallel, both compounds exhibited very low toxicity on *C. carnea*. Overall, these findings support that Mg-Chl and Mg-Chl–ZnO NC could be used as alternative approaches to synthetic pesticides for the management of *S. frugiperda*.

## Introduction

The fall armyworm (FAW) (*Spodoptera frugiperda* (J. E. Smith)) is a major invasive insect pest that affects cereal crops^1^. FAW has become a serious global threat to maize production with yield loss 67% due to its high reproductive potential and dispersal capacity^2–4^. It is predicted that FAW will cause even greater economic losses to agricultural crops since it is expected to expand its habitat^5,6^. *Spodoptera frugiperda* was officially first recorded in Egypt in 2019, having entered through the country’s southern borders of Africa, specially Sudan^7^. Because of climatic changes the fall armyworm is considered a newly emerging invasive pest of maize in Egypt^8^. Additionally, *S. frugiperda* is well known for developing resistance to insecticides^9^. In addition to decreasing the effectiveness of chemical controls, this resistance makes pest management more difficult by requiring greater dosages or more frequent application, which may have detrimental effects on the environment and human health^10^. These challenges underscore incorporating more sustainable, environmentally friendly alternatives for FAW management.

Nanotechnology could become a valuable innovation in modern agriculture, offering novel approaches for crop protection through the development of nanostructured agrochemicals^11^. Using nanoparticle formulations is one possible strategy for safeguarding agricultural crops^12,13^. High surface-to-volume ratios, high reactivity, and enhanced catalytic and biological activities are some of the unique properties of nanoparticles^14,15^. They have the capacity to damage insects by breaking through their cell membranes^16,17^. Nanoparticles, particularly metallic ones like silver (AgNPs), copper oxide (CuO NPs), and zinc oxide (ZnO NPs), could induce oxidative stress in insect cells by generating reactive oxygen species (ROS), which damage the pathogen’s proteins, lipids, and DNA^18^. Thus, one of the promising emerging approaches involves the use of natural photosensitizers, which depend on excitation of their triplet state and subsequently reaction with molecular oxygen to produce ROS, particularly a singlet oxygen (^1^O_2_) upon absorbing light. The generated reactive oxygen species (ROS) trigger profound oxidative stress in the target larvae, leading to irreversible cellular damage^19–21^. Recently, chlorophyll derivatives have been evaluated as potent photosensitizing agents for the management of agricultural and medical pests^22–24^. Such derivatives present many advantages: they are derived from natural inexpensive plant sources, approved by the Food and Drug Administration (FDA) as a food additive^25–27^. Such sensitizers are considered promising and environmentally safe alternatives to conventional pesticides for control of *Spodoptera* sp. Photosensitizers have been tested against agricultural pests, including methylene blue, rhodamine B, and rose Bengal, all of which induced significant larval mortality after ingestion and sunlight exposure^28,29^.

The integration of photosensitizers with nanoformulations, e.g., Mg-Chl–ZnO nanocomposite over bulk materials, provides many benefits. First, the high surface-to-volume ratio of nanoparticles improves the loading ability and bioavailability of chlorophyllin molecules, allowing for more effective ingestion by larvae. Second, the ZnO matrix effectively protects the photosensitizer against premature environmental degradation, thus maintaining its residual activity under field conditions. Moreover, ZnO is already a semiconductor, and this characteristic may be used in combination with chlorophyllin itself to possibly enhance the reactive ROS generation upon light exposure. This nanoformulation-based approach not only enhances the active agent-targeted delivery to insect midgut but also leads to a large cut in dosage, thereby reducing possible non-specific effects on non-target organisms.

Consequently, insects have evolved complex detoxification systems that enable them to cope with a wide range of natural toxins and substances. Two of the most important enzyme families among these systems are glutathione *S*-transferases (GSTs) and carboxylesterases (CXEs). GSTs enhance the solubility of these agents, enabling easier elimination from the body by hydrolyzing ester bonds^30^. They are directly involved in insecticide resistance by metabolizing or sequestering insecticides^31^ or indirectly by reducing oxidative stress resulting from insecticide exposure^32^. In *S. frugiperda*, epsilon GSTs have been shown to play a role in insecticide detoxification^33^. In addition to the GSTs group, CXEs represent another primary group of detoxification enzymes. They are expressed not only in insects but also in vertebrates, bacteria, and plant cells. CXEs’ main function is to hydrolyze toxic acidic esters into nontoxic subunits, such as alcohols and acids^34^.

Therefore, our work aims to introduce environmentally safe bioinsecticides as Mg-Chl and Mg-Chl/ZnO-NC to control the spread of *Spodoptera frugiperda* as a serious pest to our agricultural yield through studying the toxicity of Mg-Chl and Mg-Chl/ZnO-NC on the larval stage of *S. frugiperda* and their impact on the detoxification enzymes activity. In addition, in silico molecular docking was employed to explore the potential interaction of Mg-Chl with either *CXE22* or *SfGSTe9* and detecting their binding affinity to this compound. Thus, the adverse effect of tested chlorophyll derivatives against *Chrysoperla carnea* larvae as a non-target predator will also be evaluated.

## Materials and methods

### Chemical compounds

Tri-Sodium Magnesium Chlorophyllin (C_34_H_30_MgN_4_O_6_Na_3_) was obtained from Innovative Research and Development Incorporation (INRAD), Egypt.

All chemical reagents used in this study were of analytical grade. *Zinc acetate dihydrate* (CAS No. 5970-45-1) was purchased from *Merck* (Germany). *Sodium hydroxide* (CAS No. 1310-73-2) and *methanol* (CAS No. 67-56-1) were obtained from *Sigma-Aldrich*. *Tri-Sodium Magnesium Chlorophyllin* (CAS No. 11015-37-5) was provided by *Innovative Research and Development Incorporation (INRAD)*, Egypt. Double-distilled water (CAS No. 7732-18-5) was used for the preparation of all solutions.

### Nanomaterial synthesis

**Fig. 1:**
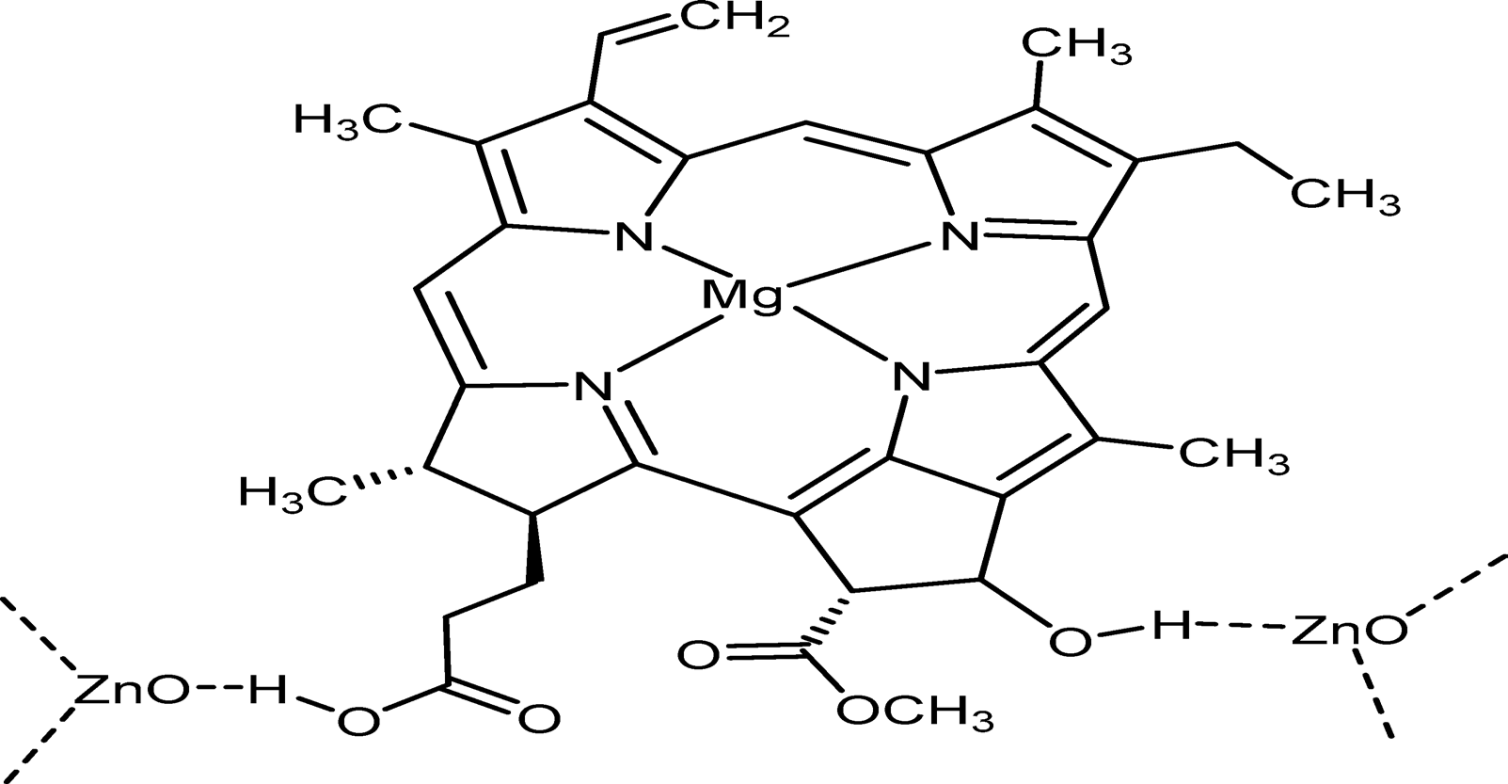
Zinc oxide nanoparticles using magnesium chlorophyllin (Mg-Chl) as a cap.

Zinc oxide nanoparticles were synthesized via a controlled reflux-assisted precipitation method. Briefly, 20 mg of zinc acetate dihydrate was dissolved in 90 mL of distilled water under magnetic stirring. The solution was refluxed at 65 °C for 2 h (optimized time; see below) to ensure complete precursor dissolution and homogeneous nucleation (Fig. 1).

Separately, 400 mg of magnesium chlorophyllin was dissolved in 10 mL of distilled water and added dropwise (0.5 mL/min) to the zinc acetate solution under continuous stirring. The reaction mixture was maintained at 65 °C for an additional 6 h to promote interaction between Zn^2+^ ions and chlorophyllin molecules.

The pH of the reaction medium was then gradually adjusted to 7.7 using 1 M NaOH under continuous stirring. The formation of ZnO nanoparticles was indicated by the appearance of turbidity due to ZnO precipitation. The final suspension was stirred for an additional 30 min to ensure reaction completion^35^.

### Characterization of prepared nanomaterials

#### UV–Vis spectra

The production of ZnO-NPs and Mg-Chl/ZnO-NCs in distilled water was monitored using a Shimadzu spectrophotometer through UV–Vis spectroscopy. The UV–Vis spectra were recorded over a wavelength range of 400–700 nm^36^.

#### Transmission electron microscopy (TEM)

The morphology and particle size of ZnO-NPs and Mg-Chl/ZnO-NC were examined using high-resolution transmission electron microscopy (HR-TEM) with a JEOL JEM-2100 instrument. For TEM analysis, samples were prepared by placing a drop of the nanoparticle suspension onto a 400-mesh carbon-coated copper grid and allowing the solvent to evaporate at room temperature.

The preservation of ZnO crystallinity after functionalization, together with the nanoscale dimensions observed by TEM and the optical modifications evidenced by UV–Vis spectroscopy, supports the formation of a magnesium chlorophyllin-functionalized ZnO hybrid nanostructure rather than a simple physical mixture.

#### Insect cultures

##### *Spodoptera frugiperda*

The *S. frugiperda* strain was obtained from Faculty of Agriculture, Cairo University. The culture was maintained for more than six generations under controlled laboratory conditions at 27 ± 1 °C, with a photoperiod of 16:8 h (L:D) and 60 ± 5% relative humidity, without exposure to pesticides. The larvae were fed daily on castor bean (*Ricinus communis* L.) leaves^10^.

##### *Chrysoperla carnea*

*Chrysoperla carnea* was obtained from the Plant Protection Research Institute, Agricultural Research Center, Dokki, Giza, Egypt. The colony was raised under controlled conditions at 22 ± 2 °C and 60–70% R.H., with a photoperiod of 12-h dark and 12-h light, and fed on nymphs of *Aphis gossypii* collected from the same location. The aphids were maintained under controlled laboratory conditions at 25 ± 2 °C, 60–70% relative humidity, with an 8-h dark period and a 16-h light period, according to Liu et al*.*^37^.

### Comparative toxicity of Mg-Chl and Mg-Chl–ZnO nanocomposite

Serial dilutions of Mg-Chl and Mg-Chl/ZnO-NC were prepared (ranging from 1.896 to 0.237 mg L^−1^). A total of 60 s-instar larvae of *S. frugiperda* were utilized for each treatment, divided into three replicates of 20 larvae per glass jar. The larvae were provided with castor bean leaves treated via the leaf-dipping method using Mg-Chl and Mg-Chl/ZnO-NC formulations. Following an initial dark incubation period of 6 and 12 h to allow for ingestion, the jars were exposed to direct sunlight for 5 h. Mortality was subsequently recorded, and lethal concentration (LC) values were determined based on the observed data. As shown in Eq. (1), the toxicity index (Sun)^38^ demonstrates the lethal concentrations, LC_50_ or LC_90_ of each compound compared with the most toxic reference compound. In Eq. (2), the relative potency represents the lethal concentrations, LC_50_ or LC_90_ of each compound compared with the least potent reference compound.

### Calculating toxicity index and relative potency

1 $${\mathrm{Sun}}^\prime{\text{s toxicity index}} = \frac{{{\mathrm{LC}}_{{50}} \; {\text{or LC}}_{{90}} \; {\text{of the most toxic insecticide}}}}{{{\mathrm{LC}}_{{50}} \; {\text{or LC}}_{{90}} \; {\text{of the other tested insecticides}}}}$$

2 $${\text{Relative potency}} = \frac{{{\mathrm{LC}}_{{50}}\; {\text{or LC}}_{{90}}\; {\text{for the least used insecticide}}}}{{{\mathrm{LC}}_{{50}}\; {\text{or LC}}_{{90}}\; {\text{of the other tested insecticides}}}}$$

### Safety profile on non-target predatory species

The calculated lethal values (LC_25_, LC_50_, LC_90_) of Mg-Chl and Mg-Chl/ZnO-NC were used to spray 20 larvae of *Chrysoperla carnea* with dark incubation for 6 and 12 h. Sprayed larvae were exposed to light for 5 h. A second trial was done to estimate the effect of tested compounds against *Chrysoperla carnea* through indirect feeding on the treated prey *Aphid gossypii,* which had previously fed on castor leaves dipped in different concentrations of Mg-Chl and Mg-Chl/ZnO-NC. Nymphs were introduced to *Chrysoperla carnea* as food, followed by dark incubation for 6 and 12 h, then exposed to light, and mortality readings were recorded after 5 h.

### Preparation of samples for enzymatic assays

Target larvae of *S. frugiperda* treated with the values of Mg-Chl and Mg-Chl/ZnO-NC were collected for biochemical analysis. The samples were homogenized in 850 µL of 0.1 M phosphate buffer (pH 7.4) using a manual glass-Teflon homogenizer (Thomas Scientific, USA). To ensure enzyme stability, the homogenization process was conducted in an ice bath. The resulting homogenates were then subjected to centrifugation at 8000 rpm for 15 min at 4 °C using a refrigerated centrifuge (Eppendorf 5417R, Germany). Following centrifugation, the pellets were discarded, and the supernatants, designated as enzyme extracts, were collected and stored at − 20 °C for subsequent enzymatic measurements.

### Biochemical assessment of glutathione *S*-transferase enzyme activity

GSTs activity was determined by measuring the conjugation of reduced glutathione (GSH) with 1-chloro-2,4-dinitrobenzene (CDNB) as a substrate at 340 nm according to the entomological protocol. The reaction mixture contained 100 µL of GSH (5 mM), 50 µL of CDNB (1 mM), and 20 µL of enzyme extract. After incubation at 30 °C for 5 min, the formation of *S*-(2,4-dinitrophenyl)-l-glutathione was monitored using a Spectronic 1201 spectrophotometer (Milton Roy Co., USA) against a reagent blank.

### Biochemical assessment of carboxylesterase enzyme activity

CXEs activity was determined using α-naphthyl acetate as a substrate. Briefly, 20 µL of the enzyme extract was incubated with 1 mL of α-naphthyl acetate (0.5 mM) at 37 °C for 30 min. The reaction was terminated by adding 50 µL of diazonium dye to facilitate the formation of a colored complex with the produced α-naphthol. The absorbance was subsequently measured at 515 nm using a Spectronic 1201 spectrophotometer (Milton Roy Co., USA).

### Molecular docking

The enzyme sequences were retrieved from UniPort (https://www.uniprot.org/). These sequences were used to generate 3D models using the AlphaFold Protein Structure Database (https://alphafold.ebi.ac.uk). The predicted 3D structures were downloaded in PDB format and subsequently converted to PDBQT format using AutoDockTools version 1.5.7, which was also employed for receptor and ligand preparation. Ligand structures were downloaded from the PubChem database. Ligands were energy-minimized prior to docking and then converted to 3D format. Docking simulations were carried out using AutoDock, which applies the Lamarckian Genetic Algorithm for efficient exploration of binding conformations. The receptor structures were treated as rigid, while ligand torsions were allowed full flexibility^39^. We used MAFFT (https://mafft.cbrc.jp/alignment/software/) to identify the conserved catalytic residues in *S. frugiperda* carboxylesterase *CXE22* (UniProt A0A5Q0TZ00) by aligning its amino acid sequence against the crystal structure of *Lucilia cuprina* esterase (PDB ID: 5TYM). The alignment confirmed the presence of the catalytic triad residues SER–GLU–HIS in *S. frugiperda*. The putative ligand-binding pocket was defined by structural alignment of the predicted receptor model to their corresponding homologous crystal structures *L. cuprina* esterase (PDB ID: 5TYM) and Epsilon-class glutathione *S*-transferase of *Anopheles gambiae* (PDB ID: 2IMI) for carboxylesterase *CXE22* and *S. frugiperda* glutathione *S*-transferase *SfGSTe9* (UniParc ID: UPI003D4B79A1), respectively. The docking grid was centered on the superimposed catalytic pocket to ensure physiologically relevant binding predictions. Post docking, the top-ranked binding poses were selected based on binding free energy and interaction stability. The amino acid residues involved in Mg-Chl binding were identified and visualized using the Protein–Ligand Interaction Profiler (PLIP) web server (https://plip-tool.biotec.tu-dresden.de/plip-web/plip/index).

### Statistics

The correlation between the mortality percentage of *S. frugiperda* larvae and compound concentration was analyzed through Probit analysis. The values for slope, LC_50_, and LC_90_ were displayed. Significant differences in mortality between treatments and enzyme assays were statistically evaluated by one-way ANOVA employing IBM SPSS Statistics. The normality of continuous variables was established using the Shapiro–Wilk test. Post-hoc multiple comparisons were performed using the least significant difference (LSD) test, and differences were considered statistically significant at *P* < 0.05.

## Results

### Morphological and structural analysis of ZnO-NPs and Mg-Chl/ZnO-NC

**Fig. 2:**
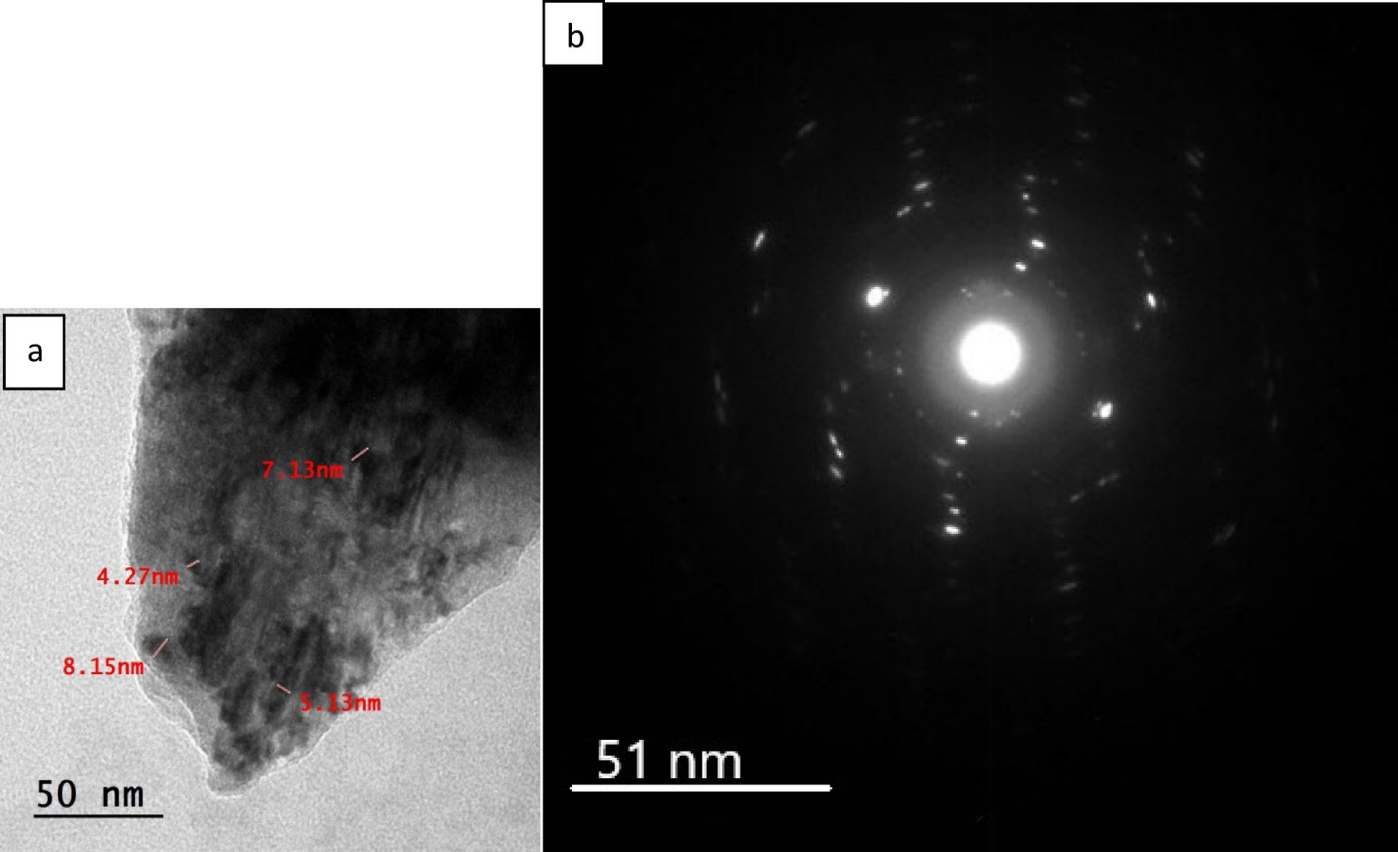
(**a**) A TEM image for Mg-Chl/ZnO-NC and (**b**) the SAED pattern showing their nanoscale size.

Transmission electron microscopy (TEM) and selected area electron diffraction (SAED) were employed to investigate the morphology and crystalline structure of the synthesized ZnO-NPs. The TEM micrograph (Fig. 2a) shows that the nanoparticles are well dispersed, with particle sizes ranging from 4.27 to 8.15 nm, confirming the formation of ultrasmall Mg-Chl/ZnO-NC. The corresponding SAED pattern (Fig. 2b) exhibits distinct concentric diffraction rings, which can be indexed to the crystallographic planes of hexagonal wurtzite structure of ZnO, demonstrating the polycrystalline nature of the nanoparticles. The presence of intense diffraction spots superimposed on the rings further validates their high crystallinity. Overall, the nanoscale dimensions observed by TEM and the pronounced crystallinity evidenced by SAED demonstrate the successful synthesis of ZnO-NPs with well-defined structural and morphological characteristics effective for biological and optical applications.

### UV–visible spectroscopy

**Fig. 3:**
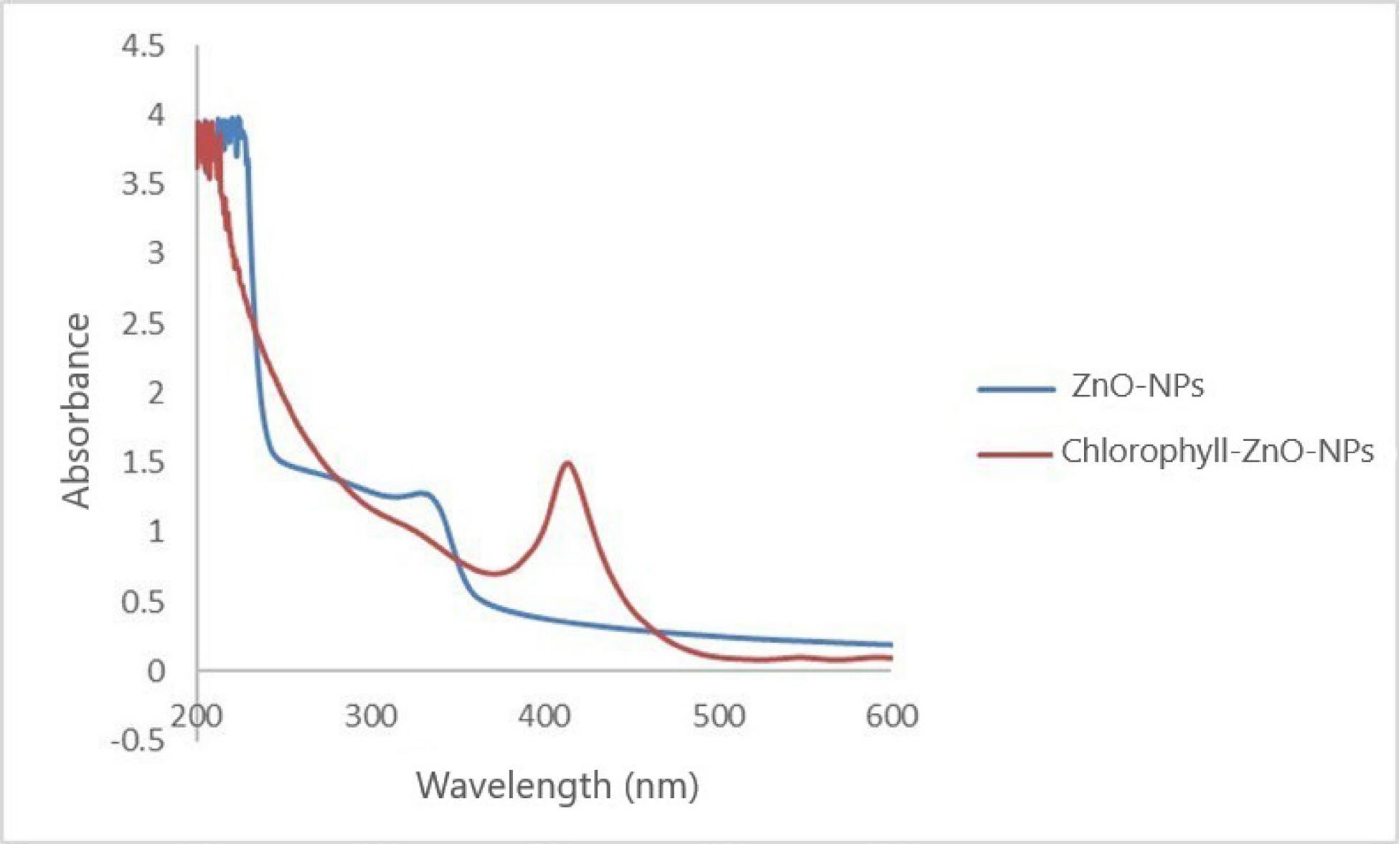
Absorbance spectral curves for synthesized ZnO-NPs and Mg-Chl/ZnO-NC.

The UV–Vis absorption spectrum of the synthesized ZnO nanoparticles (ZnO-NPs) (Fig. 3) shows a characteristic broad absorption band in the UV region, with a strong absorption peak below 300 nm. This feature validates the successful formation of nanosized ZnO and is attributed to the intrinsic band-gap transition of ZnO. The gradual tailing of the absorption edge toward the visible region proposes the presence of surface states and defect-related transitions, which are detected in metal-oxide nanoparticles.

Upon loading Mg-Chl onto the ZnO-NPs, evident changes in the UV–Vis spectrum were detected. While the intense UV absorption below 300 nm was mentained, an additional pronounced absorption band was observed in the 400–430 nm region. This new band corresponds well with the characteristic Soret band of Mg-Chl, showing the successful incorporation or adsorption of Mg-Chl molecules onto the ZnO-NPs surface. Furthermore, the red shift and increased absorbance in the visible region provide further evidence of interactions between Mg-Chl and ZnO-NPs, probably resulting from coordination between the ZnO surface and the porphyrin ring of Mg-Chl.

Broadly, the distinct spectral differences between bare ZnO-NPs and Mg-Chl–loaded ZnO-NPs support effective loading and strong interfacial interactions. The emergence of chlorophyll-specific visible absorption bands, including slight changes in the UV absorption profile, reflects potential electronic coupling between ZnO and Mg-Chl. These results show that ZnO-NPs serve as an effective carrier matrix, improving the optical properties and light-harvesting of the resulting Mg-Chl\ZnO-NC.

### Susceptibility of *Spodoptera frugiperda* larvae to nanocomposite

Laboratory bioassays were conducted to evaluate the larvicidal activity of Mg-Chl and Mg-Chl/ZnO-NC against the second-instar larvae of *Spodoptera frugiperda*. Larvae were exposed to the treatments following a 6-h incubation period and 5 h of sunlight exposure. So, the estimated LC_25_, LC_50_, and LC_90_ values for Mg-Chl were (0.29, 0.59, and 1.23 mg L^−1^), while Mg-Chl/ZnO-NC exhibited an LC_25_ of 0.24 mg L^−1^, LC_50_ value of 0.49 mg L^−1^, and LC_90_ of 1.07 mg L^−1^ (Table 1).

**Table 1:** Susceptibility status of the second larval instar of *Spodoptera frugiperda* to Mg-Chl and Mg-Chl/ZnO-NC after 6-h incubation in dark and 5-h sunlight exposure.

Concentration (mg L^−1^)	Dyes			
	Chl-Mg		Mg-Chl/ZnO-NC	
	Dead larvae	Mortality percentage	Dead larvae	Mortality percentage
0.00	0.00^a^ (60)	0.00	0.00^a^ (60)	0.00
0.237	15^b^ (60)	25.00%	18^b^ (60)	30%
0.474	24^c^ (60)	40.00%	28^c^ (60)	46.67%
0.948	36^d^ (60)	60.00%	40^d^ (60)	66.67%
1.896	53^e^ (60)	88.33%	55^e^ (60)	92%
LC_25_	0.29		0.24	
LC_50_	0.59		0.49	
LC_90_	1.23		1.07	
Slope	1.96 ± 0.28		2.06 ± 0.29	
X^2^	1.28		2.35	
*P* value	0.05		0.03	

The same letters are not significantly different, while the different letters are significantly different at *P* ≤ 0.05.

Subsequent to 12 h of dark incubation, the LC_25_ for Mg-Chl decreased to 0.26 mg L^−1^, while the LC_50_ decreased slightly to 0.51 mg L^−1^ and the corresponding LC_90_ also dropped to 1.00 mg L^−1^, compared with Mg-Chl/ZnO-NC, which exhibited an LC_25_ of 0.15, LC_50_ of 0.31, and an LC_90_ of 0.59 mg L^−1^ (Table 2). Data in Table 1 indicate that the toxicity index of Mg-Chl was 83.1% of that of Mg-Chl/ZnO-NC at the LC_50_ level against *S. frugiperda*. According to relative potency levels, the toxicity of Mg-Chl/ZnO-NC was 1.2-fold compared to the toxicity of Mg-Chl against *S. frugiperda* after 6-h incubation period and 5 h of sunlight exposure. Data in Table 2 revealed that the toxicity index of Mg-Chl was 60.8% as toxic as Mg-Chl/ZnO-NC at LC_50_ value against *S. frugiperda*. Based on relative potency levels, the toxicity Mg-Chl/ZnO-NC was 1.6-fold compared to the toxicity of Mg-Chl against *S. frugiperda* after 12-h incubation period and 5 h of sunlight exposure*.*

**Table 2:** Susceptibility status of the second larval instar of *Spodoptera frugiperda* to Mg-Chl and Mg-Chl/ZnO-NC after 12-h incubation in dark and 5-h sunlight exposure.

Concentration (mg L^−1^)	Dyes			
	Chl-Mg		Mg-Chl/ZnO-NC	
	Dead larvae	Mortality percentage	Dead larvae	Mortality percentage
0.00	0.00^a^ (60)	0.00	0.00^a^ (60)	0.00
0.237	19^b^ (60)	31.67%	26^b^ (60)	43.33%
0.474	28^c^ (60)	46.67%	37^c^ (60)	61.67%
0.948	44^d^ (60)	73.33%	47^d^ (60)	78.33%
1.896	56^e^ (60)	92.50%	57^e^ (60)	95.00%
LC_25_	0.26		0.15	
LC_50_	0.51		0.31	
LC_90_	1.00		0.59	
Slope	2.04 ± 0.31		2.11 ± 0.03	
X^20^	2.22		2.41	
*P* value	0.02		0.01	

Statistical analysis demonstrated significant differences in larval mortality with increasing concentrations at *P* ≤ 0.05. Extending the dark incubation period after Mg-Chl treatment did not produce a significant change in LC_50_ values. However, in the case of Mg-Chl/ZnO-NC, increasing the dark incubation period significantly enhanced toxicity, as reflected by the reduced lethal concentration values.

### Predator susceptibility assessment to nanocomposite

The toxicity data presented in Tables 3 and 4 demonstrate that both Mg-Chl and Mg-Chl/ZnO-NC exhibit very low toxicity toward the beneficial predator *Chrysoperla carnea* under both direct and indirect exposure conditions.

**Table 3:** Effect of Mg-Chl and Mg-Chl/ZnO-NC as direct spraying after 6- and 12-h incubation in dark and 5-h sunlight exposure on non target predator *Chrysoperla carnea*.

Photosensitizer	Concentration (mg L^−1^)	Dead larvae	%M	LC_50_	LC_90_	Slope	X^2^	*P* value
6 h								
16.49	LC_25_ (0.29)	4^b^ (60)	6.67	173.33	9087.13	0.37 ± 0.44	0.97	0.34
	LC_50_ (0.59)	7^c^ (60)	11.67					
	LC_90_ (1.23)	9^c^ (59)	15.25					
16.49	LC_25_ (0.24)	5^b^ (60)	8.33	157.92	8045.01	0.40 ± 0.37	1.01	0.28
	LC_50_ (0.49)	8^c^ (59)	13.56					
	LC_90_ (1. 07)	10^c^ (60)	16.67					
12 h								
Mg-Chl	LC_25_ (0.26)	6^b^ (60)	10.00	143.96	7037.44	0.42 ± 0.47	1.22	0.24
	LC_50_ (0.51)	9^c^ (60)	15.00					
	LC_90_ (1.00)	10^c^ (59)	16.95					
Mg-Chl/ZnO-NC	LC_25_ (0.15)	7^b^ (60)	11.67	135.19	6035.53	0.48 ± 0.36	1.41	0.21
	LC_50_ (0.31)	10^c^ (59)	16.49					
	LC_90_ (0.59)	12^c^ (60)	20.00					
Control	0	0.00^a^ (60)	0.00	–	–	–	–	–

*The same letters are not significantly different, while the different letters are significantly different at *P* ≤ 0.05.

**Table 4:** Effect of Mg-Chl and Mg-Chl/ZnO-NC as indirect ingestion after 6- and 12-hourincubation in dark and 5-h sunlight exposure on non-target predator *Chrysoperla carnea*.

Photosensitizer	Concentration (mg L^−1^)	Dead larvae	%M	LC_50_	LC_90_	Slope	X^2^	*P* value
6 h								
Mg-Chl	LC_25_ (0.29)	1^b^ (60)	1.66	243.69	9223.24	0.27 ± 0.50	0.67	0.51
	LC_50_ (0.59)	3^c^ (60)	5.00					
	LC_90_ (1.23)	4^c^ (59)	6.78					
Mg-Chl\ZnO-NC	LC_25_ (0.24)	2^b^ (60)	3.33	221.02	9178.02	0.30 ± 0.49	0.72	0.47
	LC_50_ (0.49)	5^c^ (59)	8.47					
	LC_90_ (1.07)	7^c^ (60)	11.67					
12 h								
Mg-Chl	LC_25_ (0.26)	3^b^ (60)	5.00	196.11	9108.22	0.33 ± 0.50	0.75	0.41
	LC_50_ (0.51)	6^c^ (60)	10.00					
	LC_90_ (1.00)	7^c^ (59)	11.86					
Mg-Chl/ZnO-NC	LC_25_ (0.15)	4^b^ (60)	6.67	184.57	9098.17	0.35 ± 0.34	0.88	0.39
	LC_50_ (0.31)	6^c^ (59)	10.17					
	LC_90_ (0.59)	8^c^ (60)	13.33					
Control	0	0.00^a^ (60)	0.00	–	–	–	–	–

*The same letters are not significantly different, while the different letters are significantly different at *P* ≤ 0.05.

Following direct application, mortality levels remained low even at concentrations corresponding to LC_90_ values determined for the target pest. At 6 h post-treatment, LC_50_ values reached 173.33 mg L^−1^ for Mg-Chl and 157.92 mg L^−1^ for Mg-Chl/ZnO-NC, while LC_90_ values exceeded 8000 mg L^−1^, indicating a wide safety margin for the predator. A similar trend was observed after 12-h exposure, with LC_50_ values remaining exceptionally high for both formulations.

Under indirect exposure via ingestion of treated prey, toxicity was further reduced. LC_50_ values increased markedly, exceeding 240 mg L^−1^ for Mg-Chl and 220 mg L^−1^ for Mg-Chl/ZnO-NC after 6-h incubation period and remained above 180 mg L^−1^ after 12 h. Mortality percentages did not exceed 13–20% even at the highest tested concentrations.

### Enzyme activity assessment

The effects of Mg-Chl and Mg-Chl/ZnO-NC on detoxification enzymes were evaluated to better understand their mode of action. Table 5 summarizes the activities of glutathione S-transferases (GSTs) and carboxylesterases (CXEs) in *S. frugiperda* larvae treated with LC_50_ concentrations of Mg-Chl and Mg-Chl/ZnO-NC after 6 h of dark incubation followed by 5 h of sunlight exposure. GSTs activity significantly decreased to 6.79 and 6.00 µmol min^−1^ µg^−1^ protein in larvae treated with Mg-Chl and Mg-Chl/ZnO-NC, respectively, compared with 7.09 µmol min^−1^ µg^−1^ protein in the control. In contrast, CXEs activity increased significantly to 1468.33 and 1691.00 µmol min^−1^ µg^−1^ protein following treatment with Mg-Chl and Mg-Chl/ZnO-NC, respectively, relative to 1224.33 µmol min^−1^ µg^−1^ protein in the control.

**Table 5:** Glutathione *S*-transferases and carboxylesterases activity variations after treatment of *Spodoptera frugiperda* with LC_50_ values of Mg-Chl and Mg-Chl/ZnO-NC for 6-and 12-h dark incubation period and 5-h sunlight exposure time.

Dye	GSTs (µmol/min/µg protein) and 1-chloro-2,4-dinitrobenzene (CDNB) as a substrate		Carboxylestrases (µmol/min/µg protein) and α-naphthyl acetate as a substrate	
	6 h	12 h	6 h	12 h
Control	7.09 ± 0.18^a^	6.71 ± 0.15^a^	1224.33 ± 13.05^a^	244.67 ± 0.09^a^
Mg-Chl	6.79 ± 0.18^b^	5.74 ± 0.13^b^	1468.33 ± 10.41^b^	292.67 ± 0.15^b^
Mg-Chl/ZnO-NC	6.00 ± 0.17^c^	5.00 ± 0.12^c^	1691.00 ± 22.87^c^	323.00 ± 0.18^c^

Data are presented as mean ± SE. Means were analyzed by ANOVA test; at *P* ≤ 0.05, means followed by the same letters are not significantly different, while means followed by the different letters are significantly different.

After 12 h of dark incubation followed by 5 h of sunlight exposure, a similar trend was observed. GSTs activity decreased significantly to 5.74 and 5.00 µmol min^−1^ µg^−1^ protein in Mg-Chl and Mg-Chl/ZnO-NC treated larvae, respectively, compared with 6.71 µmol min^−1^ µg^−1^ protein in the control. Conversely, CXEs activity increased significantly to 292.67 and 323.00 µmol min^−1^ µg^−1^ protein after treatment with Mg-Chl and Mg-Chl/ZnO-NC, respectively, compared with 244.67 µmol min^−1^ µg^−1^ protein in the control. These results indicate that both formulations modulate key detoxification enzymes in *S. frugiperda*, with enhanced effects observed for the nanocomposite based formulation.

**Fig. 4:**
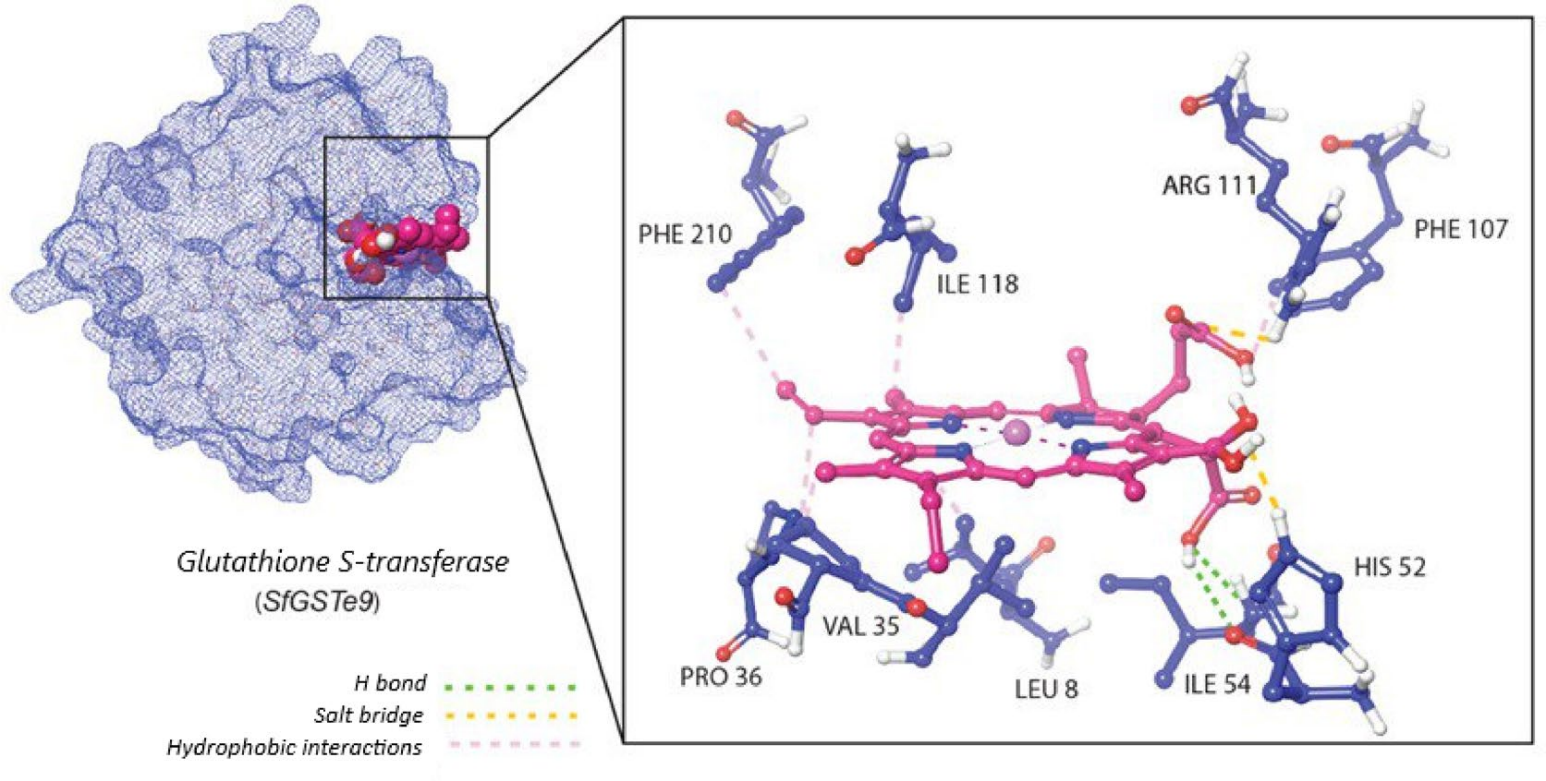
Homology model of *S. frugiperda* GST and Mg-Chl. Left: surface representation of *S. frugiperda SfGSTe9* (blue) with Mg-Chl (pink) docked in the binding pocket. Right: detailed interactions between Mg-Chl and GST residues; hydrogen bonds with HIS52 and ILE54 are shown, along with hydrophobic contacts and salt bridges (dashed lines). Binding free energy =  − 13.53 kcal/mol.

Insecticide bioassays, together with the observed alterations in CXEs and (GSTs) activities, motivated homology modeling and molecular docking analyses of these enzymes with magnesium chlorophyllin (Mg-Chl). Docking simulations positioned Mg-Chl within the hydrophobic H-site of the *SfGSTe9* enzyme, where the ligand formed hydrogen bonds with HIS52 and ILE54, in addition to stabilizing hydrophobic interactions (Table 6; Fig. 4). Consistent with the in vivo reduction in GSTs activity following Mg-Chl exposure, the docking results revealed a strong predicted binding affinity (− 13.53 kcal/mol). This suggests that Mg-Chl may function as an antagonist against GSTs by occupying the active site and restricting access to the native substrate.

**Table 6:** Amino acid residues in predicted 3D enzyme structures interacting with Mg-Chl.

Enzyme	H bonds	Hydrophobic interaction	Salt bridge
*SfGSTe9*	HIS52, ILE54	LEU8, VAL35, PRO36, PHE107, ILE118, PHE210	HIS52, ARG111
*CXE22*	ARG85, ASN80, SER284	THR125, LEU128, VAL281, VAL344, PHE448	ARG85

**Fig. 5:**
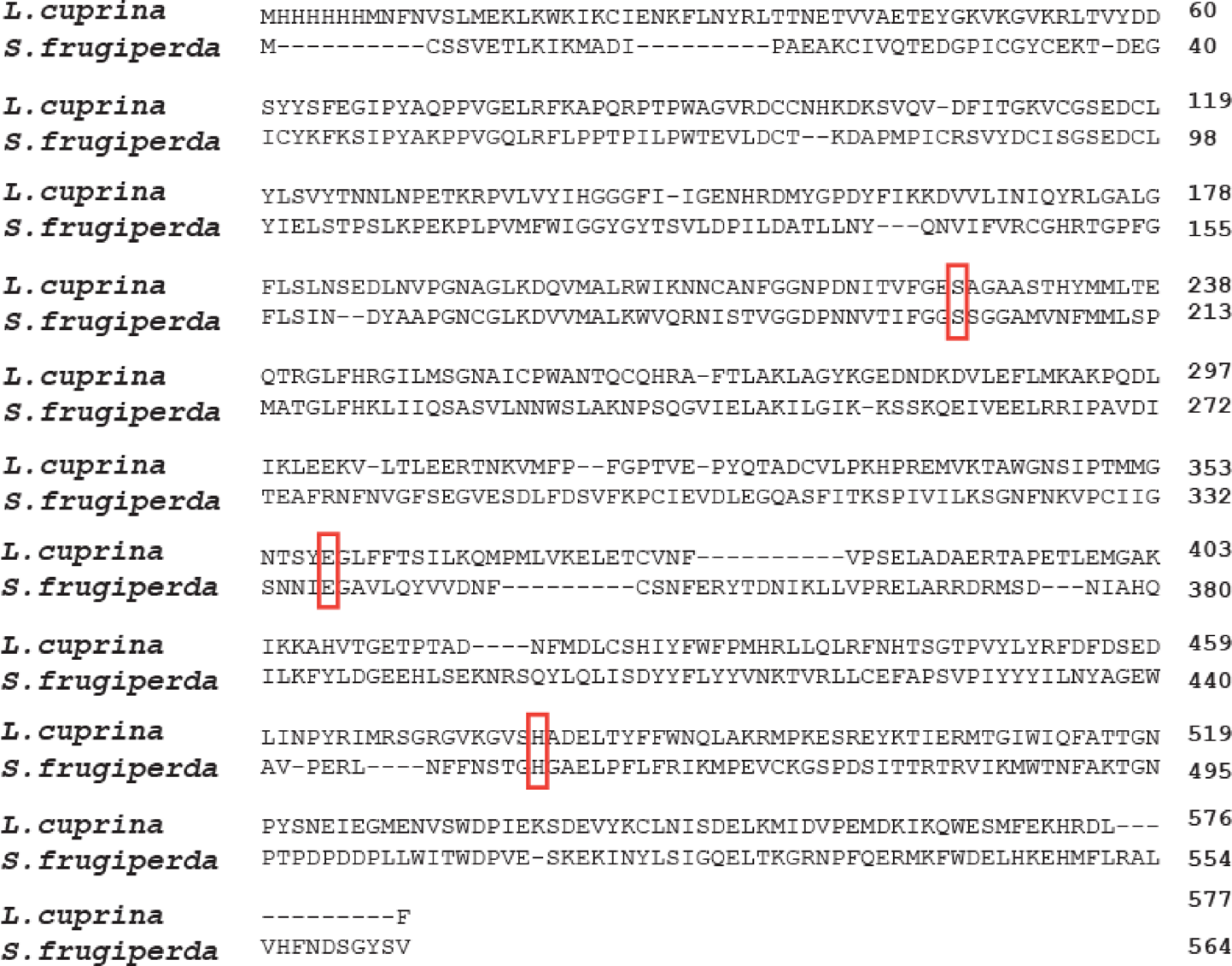
Amino acid sequences alignment between *L. cuprina* esterase and *S. frugiperda* carboxylesterase *CXE22*. Conserved catalytic triad residues (SER[S], GLU[E], HIS[H]) are highlighted (red boxes).

**Fig. 6:**
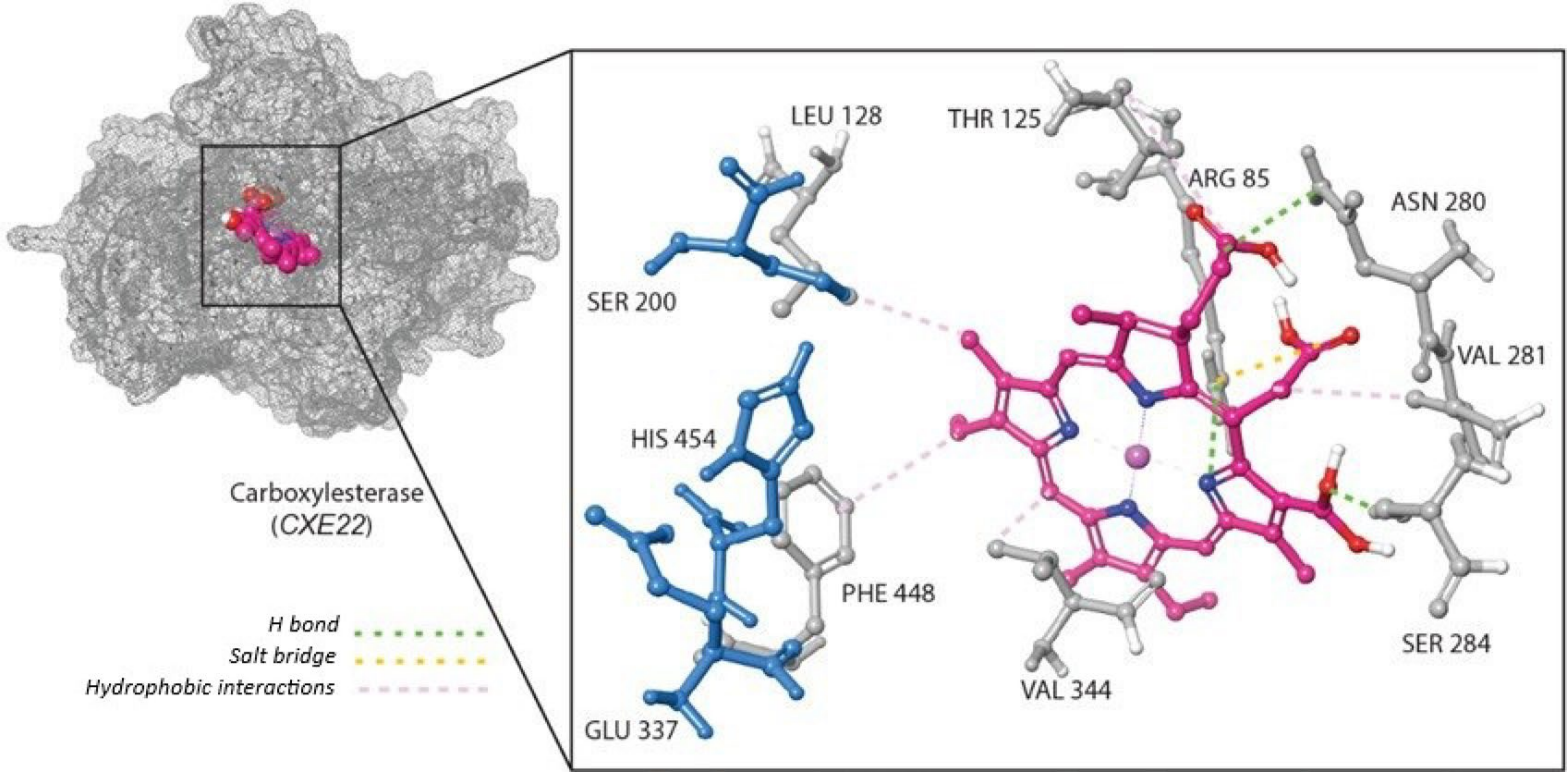
Homology model of *S. frugiperda* carboxylesterase and Mg-Chl. Left: surface representation of *S. frugiperda* carboxylesterase *CXE22* (gray) with Mg-Chl (pink) in the binding pocket. Right: detailed interactions between Mg-Chl and *CXE22*; hydrogen bonds with ARG85, ASN280, and SER284 are shown, along with hydrophobic contacts and salt bridges (dashed lines). The catalytic triad SER200, GLU337, and HIS454 are shown in cyan color. Binding free energy =  − 4.61 kcal/mol.

For the carboxylesterase *CXE22* model, sequence alignment with *Lucilia cuprina* esterase confirmed the presence of the conserved catalytic triad (SER–GLU–HIS) in *S. frugiperda* (Fig. 5). Mg-Chl was also predicted to dock within the *CXE22* binding pocket, forming hydrogen bonds with ARG85, ASN280, and SER284, along with additional hydrophobic interactions (Table 6; Fig. 6). However, the predicted binding free energy (− 4.61 kcal/mol) was notably weaker than that observed for *SfGSTe9*. Nonetheless, the interaction of Mg-Chl with residues surrounding the conserved catalytic triad suggests a potential interference with carboxylesterases catalytic function. Taken together, our predicted docking models reinforce the claim that Mg-Chl has a toxicological effect on *S. frugiperda* control.

## Discussion

Interest in the use of the chlorophyll derivatives as photosensitizing agents for pest management in agriculture is rising due to the characteristics of being biodegradable and environmentally safe, along with low toxicity to humans and other non-target organisms^40,41^. Unlike standard synthetic pesticides, which often lead to an imbalance in ecosystems and also exist as bioaccumulative chemicals within an environment, photosensitizers provide an environmentally friendly and sustainable approach. There are many materials available for use in developing photosensitizers (natural as well as synthetic), and both porphyrins and chlorophyll derivatives have demonstrated effective insecticidal components by producing ROS during exposure to light^42,43^. Magnesium chlorophyllin is one of the more commonly used water-soluble chlorophyll derivatives that has been documented as producing powerful photodynamic effects on numerous insect pests and aquatic parasites due to the ability to produce singlet oxygen when exposed to light^44^.

Spectroscopic characterization via UV–Vis confirmed the successful formation and functionalization of ZnO nanoparticles with Mg-Chl. The intrinsic band-gap transition observed below 300 nm is due to quantum confinement effects, while the extension of absorption into the visible range shows the presence of defect-based electronic transitions, which are important for enhanced photocatalytic and photodynamic activity. Following Mg-Chl loading, a distinct Soret band appeared between 400 and 430 nm, followed by a noticeable red shift. These spectral alterations suggest strong electronic interactions likely through electrostatic bonding between the Mg-Chl porphyrin ring and the ZnO surface. This synergistic integration significantly enhances light harvesting and improves the production of reactive oxygen species (ROS), thereby causing the potent oxidative stress-mediated mortality noticed in *S. frugiperda* larvae.

In the present study, Mg-Chl exhibited considerable toxicity against *S. frugiperda*, and its efficacy increased with both concentration and dark incubation period prior to light exposure, consistent with earlier reports^45–47^. However, Mg-Chl/ZnO-NC showed higher toxicity than Mg-Chl alone. Based on LC_50_ values, Mg-Chl/ZnO-NCs were 1.2-fold more toxic after 6 h of incubation in dark and 1.6-fold more toxic after 12 h, highlighting the increasing efficacy of the nanoform. We assumed that the relatively small difference in mortality rate between Mg-Chl and its ZnO-loaded counterpart at certain concentrations may be involved in inherently photodynamic activity of chlorophyllin, which can lead to saturation of toxic effects. Also, the high toxicity of Mg-Chl/ZnO-NC is a result of many synergistic biochemical and physicochemical mechanisms. Both ZnO nanoparticles and chlorophyllin consider strong photosensitizers after sunlight exposure, which enhance photodynamic activity through increased ROS generation. The nanoscale dimensions of ZnO allow for easier penetration through the integument and gut epithelium of the insect, thus increasing the amount of active ingredient that can be taken up by the insect cell. The immobilization of chlorophyllin onto ZnO-NPs increases the photostability of chlorophyllin, while extending the time that it can exhibit biological activity^48^. In addition, the synergistic nature of the combined formulation strongly affects detoxification pathways by causing a significant reduction in GST activity and an increase in carboxylesterase activity to the extent that it depletes the antioxidant defense system of the insect^49,50^. The intrinsic toxicity associated with ZnO-NPs, including the induction of oxidative stress, membrane damage, and mitochondrial dysfunction, adds to the overall insecticidal activity of the combined formulations^51^. These interdependent interactions between Mg-Chl/ZnO-NC formulations contribute to their significantly greater toxicity than that of the individual component of Mg-Chl and demonstrate their potential to be an effective and ecologically sound method of controlling *S. frugiperda.* We suppose that the remarkable selectivity of chlorophyllin between the target larvae and the non-target predator *C. carnea* can be attributed to several factors. First, cuticle thickness plays a vital role; the robust, sclerotized integument of *C. carnea* likely acts as a superior physical barrier against the penetration of the photosensitizer compared to the thinner procuticle of *S. frugiperda* larvae. Second, the enzymatic defense system of *C. carnea* might be more efficient in neutralizing ROS generated by the photodynamic action, providing a higher threshold of protection against oxidative stress.

The current findings align with the earlier studies from Kumaravel et al.^52^ and Ruiz-Aguilar et al.^53^, which have all indicated that photosensitizers and their nanoformulations can impact the insect detoxification systems. In the present study, the effects of Mg-Chl and Mg-Chl/ZnO-NC, at LC_50_ concentrations, on glutathione *S*-transferases (GSTs) and carboxylesterases (CXEs) activity, in second-instar *S. frugiperda* larvae subjected to a 6-h dark incubation period and a subsequent 5-h exposure to sunlight. The generated data support significant elevations in carboxylesterase activity for both formulations, as compared to the control, with concurrent significant declines in GST activity levels. We observed similar enzymatic response profiles at 12 h of dark incubation to confirm that both formulation treatments resulted in biochemically consistent and time-dependent effects. This explained that larval mortality is due to the massive surge of reactive oxygen species (ROS) and the resulting irreversible oxidative damag^23^ rather than enzymatic alterations.

The observed increase in esterase activity is consistent with previous research that reported that *Spodoptera* spp. show an elevation in their esterase levels following treatment with insecticides. Specifically, Magdy et al.^54^ noticed increased esterase activity in *S. littoralis* larvae treated with Spinosad. Abd el-Mageed et al.^55^ reported an increase in the esterase activity of *S. littoralis* fourth-instar larvae treated with LC_50_ concentrations of the same compound. Esterases are key phase I detoxification enzymes involved in the metabolism of a wide range of xenobiotics, including natural and synthetic insecticides^56^. Their increased activity is often interpreted as an adaptive response that enhances tolerance to toxic compounds through accelerated hydrolysis and detoxification^57^. Carboxylesterases catalyze the hydrolysis of ester bonds, converting insecticides into less toxic and more water-soluble metabolites via a catalytic serine-dependent process^58^. This hydrolytic pathway represents an important defense mechanism by which insects overcome chemical stress^59,60^.

In contrast, GSTs activity in the larvae exposed to Mg-Chl and Mg-Chl–ZnO-NC was significantly suppressed, which is similar to the findings from *S. littoralis*, according to Muthusamy et al.^61^. In their study, *S. littoralis* larvae had GSTs activity after being treated with Pyrethroids and Organophosphates. GSTs are responsible for Phase II detoxification by catalyzing the conjugation of reduced glutathione (GSH) onto the toxic substances, thus enhancing the solubility of the toxicants, facilitating better excretion^62^. Reductions in GSTs activity generally indicate a depletion of GSH as a result of oxidative stress; GSH is used to neutralize reactive oxygen species^63^. Additionally, some toxicants may interact with the active sites of GSTs causing an inhibition in their activity or changes in the conformation of the GSTs resulting in a loss of enzymatic activity^64^. Also, GSTs are involved in the reduction of lipid hydroperoxides to harmless alcohols, which decreases cellular damage (due to peroxidation) and contributes to the protection of the cellular membranes, proteins and DNA^65^.

Molecular docking analyses revealed that Mg-Chl binds strongly to different amino acid residues within the hydrophobic site (H-site) of *SfGSTe9* in *S. frugiperda.* The high binding affinity (− 13.53 kcal/mol) of Mg-Chl supports the hypothesis that GSTs play a role in insecticide detoxification and oxidative stress processes in *S. frugiperda* larvae. Furthermore, strong binding interactions with HIS52 and ILE54 residues reinforce the GSTs inhibition by Mg-Chl^66^. Conventional insecticides such as emamectin benzoate and chlorantraniliprole showed similar binding with HIS52 and ARG111^67^, suggesting a common mode of action. However, the larval mortality after Mg-Chl intake suggests an inhibitory role by Mg-Chl; we believe that the large size and strong binding affinity of Mg-Chl may partially block or occupy the glutathione (GSH) binding site (G-site), affecting the GSH interaction with the GSTs enzymes. In addition, Mg-Chl might provoke cellular oxidative stress; therefore, the amount of GSH in the cells decreased. Downregulation of GSTs in *S. frugiperda* has been noticed in several insect species, such as *Locusta migratoria*^68^, *Nilaparvata lugens*^69^, *Bactrocera dorsalis*^70^, and *Cnaphalocrocis medinalis*^71^ under various stress conditions, highlighting the system complexity and the need for further studies to understand such an interesting mechanism.

Moreover, Mg-Chl is moderately bound to *S. frugiperda* carboxylesterase *CXE22* (− 4.61 kcal/mol) via multiple H bonds (ARG85, ASN280, SER284) and other interactions. However, the binding energy is weaker than the GST; the model showed a close proximity of Mg-Chl to the catalytic triad (SER, GLU, HIS) in the active site, highlighting a plausible role of Mg-Chl in provoking the CXEs activity and modulating the catalytic efficiency^72,73^. Although *S. frugiperda* expresses multiple isoforms of carboxylesterase, the docking analysis revealed that Mg-Chl moderately binds to *CXE22*. Mg-Chl might show a stronger binding with other copies of CXEs, explaining their upregulation in the larvae tissues.

In addition, the increase in the total activity of the CXEs after the larvae are treated with Mg-Chl might be a response to the oxidative stress caused by the Mg-Chl; such compensatory upregulation is a tool used by insects to accommodate the different ecological stress conditions, whereas some detoxicating enzymes such as cytochrome P450s or carboxylesterases are upregulated when some of the insect detoxification pathways are overloaded^32^. Such a phenomenon is reported before in insects such as *Spodoptera litura* and *Helicoverpa armigera*, where the enzyme upregulation crosstalk happened between GSTs and CXEs after the insecticide treatment^74^, suggesting an alternative explanation to the increase in the activity of CXEs, i.e., inhibition of the GSTs by Mg-Chl leads to increasing the oxidative stress signals inside the cells, by which the CXEs are upregulated instead of the direct interaction between CXEs and Mg-Chl.

Although both formulations exhibited very low toxicity toward *C. carnea*, Mg-Chl consistently showed higher LC_50_ and LC_90_ values and lower mortality percentages than Mg-Chl/ZnO-NC, indicating a slightly higher safety margin for the predator. These findings support their compatibility with integrated pest management (IPM) programs and their minimal risk to non-target beneficial insects.

### Limitations

Despite the significant findings, some limitations must be acknowledged. First, using natural sunlight as an activation source gives environmental variability in light intensity in comparison with artificial sources. Second, the reliance on AlphaFold-predicted enzyme models, while very accurate for docking, needs future experimental validation through crystal or cryo-EM structures. Finally, these bioassay laboratory experiments are required, yet large-scale field trials are essential to evaluate the long-term stability and effectiveness of the Mg-Chl–ZnO NCs under complicated environmental fluctuations.

## Conclusion

This study highlights Mg-Chl and Mg-Chl–ZnO NCs as sustainable alternatives to chemical insecticides for *S. frugiperda* management, suggesting a triple advantage: high larvicidal effectiveness, ecological safety for the predator *C. carnea*, and an oxidative stress based on mode of action that reduces insecticide resistance. Molecular docking and biochemical assays validated that these photosensitizers disrupt larval homeostasis by modulating key detoxification enzymes. Overall, these findings support the integration of chlorophyll-based nanocomposite into ecofriendly pest management programs.

## Data Availability

The datasets used and/or analyzed during the current study are available from the corresponding author upon reasonable request.
